# Duration of the effectiveness of nicotine electronic cigarettes on smoking cessation and reduction: Systematic review and meta-analysis

**DOI:** 10.3389/fpsyt.2022.915946

**Published:** 2022-08-04

**Authors:** Paul Vanderkam, Audrey Bonneau, Shérazade Kinouani, Palina Dzeraviashka, Philippe Castera, Marc Besnier, Philippe Binder, Nicolas Doux, Nematollah Jaafari, Claire Lafay-Chebassier

**Affiliations:** ^1^INSERM U-1084, Experimental and Clinical Neurosciences Laboratory, University of Poitiers, Poitiers, France; ^2^Clinic Research Unit, Centre Hospitalier Henri Laborit, Poitiers, France; ^3^Department of General Practice, University of Bordeaux, Bordeaux, France; ^4^Department of General Practice, Poitiers, France; ^5^Bordeaux Population Health Research Center, Univ. Bordeaux, Inserm, Team HEALTHY, UMR 1219, Bordeaux, France; ^6^Service Commun de Documentation, Bibliothèque Universitaire de Médecine et de Pharmacie, University of Poitiers, Poitiers, France; ^7^Department of Clinical Pharmacology, Poitiers University Hospital, Poitiers, France; ^8^INSERM, Clinical Investigation Center CIC 1402, University of Poitiers, CHU Poitiers, Poitiers, France

**Keywords:** electronic cigarettes (E-cigarettes), smoking cessation, smoking reduction, serious adverse effects, Electronic Nicotine Delivery Systems (ENDS)

## Abstract

**Background:**

The success of pharmacotherapies for smoking cessation in real-life remains limited, with a significant number of long-term relapses. Despite first promising results, the duration of the effectiveness of electronic cigarettes is still unknown. Our objective was to assess the duration of the effectiveness of electronic cigarettes on smoking cessation and reduction in daily smokers.

**Methods:**

The databases EMBASE, Cochrane Central Register of Controlled Trials (CENTRAL), ClinicalTrials.gov and PUBMED were consulted until March 23, 2022. We selected only randomized controlled trials with daily adult smokers. The intervention was the nicotinic electronic cigarette *vs*. non-nicotine electronic cigarette or other validated pharmacotherapies (varenicline, bupropion and nicotine replacement therapy). The minimum duration of the intervention was 3 months, with a follow-up of at least 6 months. Two independent reviewers used the PRISMA guidelines. The primary endpoint was smoking cessation at the end of the intervention and follow-up periods confirmed by a reduction in expired CO < 10 ppm. The reduction was defined as at least 50% of the initial consumption or by a decrease of daily mean cigarette consumption at the end of the intervention and follow-up periods.

**Results:**

Abstinence at the end of the intervention and follow-up periods was significantly higher in the nicotine electronic cigarette group, compared to nicotine replacement therapy (NRT) [respectively: RR: 1.37 (CI 95%: 1.32–2.93) and RR: 1.49 (CI 95%: 1.14–1.95)] and to the non-nicotine electronic cigarette condition [respectively: RR: 1.97 (CI 95%: 1.18–2.68) and RR: 1.66 (CI 95%: 1.01–2.73)]. With regard to smoking reduction, the electronic cigarette with nicotine is significantly more effective than NRT at the end of the intervention and follow-up periods [respectively RR: 1.48 (CI 95%: 1.04–2.10) and RR: 1.47 (CI 95%: 1.18–1.82)] and non-nicotine electronic cigarette in the long term [RR: 1.31 (CI 95%: 1.02–1.68)].

**Conclusions:**

This meta-analysis shows the duration of the effectiveness of the nicotine electronic cigarette *vs*. non-nicotine electronic cigarette and NRT on smoking cessation and reduction. There are still uncertainties about the risks of its long-term use and its potential role as a gateway into smoking, particularly among young people.

## Background

Each year, 8 million deaths are linked to tobacco use, including 1.2 million non-smokers involuntarily exposed to tobacco smoke (1). In addition, the morbidities caused by tobacco smoking have multiple harmful consequences and disrupt the psychological, familial and social equilibrium, with a high cost for society. It remains to be the world's leading cause of preventable death and a major economic challenge (2).

Smoking cessation is an important factor in reducing overall mortality. The earlier smokers quit, the greater the health benefits are. It is the decrease in smoking duration, rather than the decrease in the number of cigarettes smoked per day, that has the highest impact on health benefits (3).

For this purpose, several cessation aids exist. For pharmacotherapies, nicotine replacement therapy shows a 50–70% increase in the cessation rate. Compared to a placebo, varenicline doubles a smoker's chances of stopping, *and* it helps 50% more patients *than* nicotine patches and other substitutes (4). Finally, behavioral management and support associated with the various treatments increase the chances of smoking cessation by approximately 10–20% (4, 5). However, the success of these methods in real life remains limited, with a significant number of long-term relapses (6).

Developing strategies for refractory patients to make use of pharmacotherapies and for those who are not ready for complete abstinence is important. In this context, the tobacco harm reduction approach is on the rise. It involves achieving a safer alternative to tobacco consumption beyond complete smoking cessation (7). The overriding aim is to make it possible for people who are unable to stop smoking to consume nicotine in a less harmful form than tobacco (8, 9).

In this context, the nicotine electronic cigarette appeared in the 2000s (10). It is mainly composed of nicotine (optional), propylene glycol, glycerin and flavoring. It allows the inhalation of nicotine after heating the liquid. The principle is to produce an aerosol that imitates tobacco smoke by using a heating resistor that is part of the atomiser. Unlike a conventional cigarette, there is no combustion. Four generations of such devices have been marketed, and they have become increasingly effective in terms of autonomy, the distribution of nicotine and marketing (11–13).

Users generally have a good overall perception of electronic cigarettes and say that using them is a viable way of reducing or even stopping their tobacco consumption. In the context of stopping smoking, even though a majority of people try to quit alone, there has been an increase in the use of electronic cigarettes to help people stop smoking (14–16). Since emerging in the 2010s, the market for electronic cigarettes has stabilized despite the many controversies it has generated (17).

Despite a recent meta-analysis (18) clear recommendations do not exist because of the small number of studies that have been carried out and incomplete data on the effects of electronic cigarettes or their duration. Moreover, a recent study has questioned their effectiveness and noted a possible decrease in weaning since they were introduced into the European Union (19).

To update the actual knowledge on the efficacy of nicotine electronic cigarettes, we conducted a meta-analysis to answer questions about their duration of efficacy and safety.

## Methods

We conducted a systematic review following the Preferred Reporting Items for Systematic review and Meta-Analyses (PRISMA) guidelines (20).

### Objectives

- Main: To assess the duration of the effectiveness of nicotine electronic cigarettes on smoking cessation and reduction in daily smokers.
- Secondary: To investigate the long-term safety of nicotine electronic cigarettes.

### Research method

The databases EMBASE, Cochrane Central Register of Controlled Trials (CENTRAL), ClinicalTrials.gov and PUBMED were consulted until march 23, 2022.

The following keywords/booleans were selected:

(1) for the device:

Mesh terms: *Electronic Nicotine Delivery Systems (ENDS), Electronic nicotine delivery device*

(2) for use:

Mesh terms: *Vaping, Electronic Cigarette Use, E-Cig Use, E-Cigarette Use*

(3) for quitting:

Mesh terms: *Smoking cessation, Quitting smoking, Tobacco cessation*

(4) for reduction:

Mesh terms: *Smoking reduction*

Non-Mesh terms: *Harm reduction*.

### Eligibility criteria

Randomized controlled trials with daily adult smokers (> 10 cigarettes per day) were selected.

The study population includes smokers over 18 years of age without severe unstable diseases and current pregnancy or breastfeeding, with or without the intention of quitting. The intervention was the nicotine electronic cigarette *vs*. non-nicotine electronic cigarette or other validated pharmacotherapies (varenicline, bupropion and nicotine substitutes). Among the trials, those with a minimum of 3 months of intervention and a follow-up of at least 6 months were selected.

The primary endpoint was smoking cessation at the end of the intervention and follow-up period confirmed by a reduction in expired CO < 10 ppm. We used the most rigorous definition of abstinence when it was available. On the other hand, a reduction was defined as at least 50% of the initial consumption or by a decrease of daily mean cigarette consumption at the end of the intervention and follow-up period.

The secondary endpoint was the occurrence of the reported serious adverse effects of the nicotine electronic cigarette at the end of the follow-up period. Seriousness was defined as any effect leading to hospitalization (initial or prolonged), permanent disability, life-threatening situation or death (ICH Expert Working Group).

Trials not published in English or French were excluded.

### Screening and data extraction

Studies measuring only effects on withdrawal syndrome were excluded. Two authors (AB, PV) independently screened the titles and abstracts of search hits to select studies of interest and reviewed the full texts. Disagreements were resolved by discussion between the authors. Information on methodology, participants and interventions, as well as the outcome measures, were collected by AB on an Excel spreadsheet and cross-checked by PV.

### Risk of bias

The risk of bias was calculated using the new Cochrane RoB 2 Tool for randomized trials.

### Quantitative analyses

The quantitative analyses were performed with the Revman^®^ software version 5.3. The analyses were stratified for each outcome criterion by specific intervention and by comparator.

Once the results were pooled, we calculated the relative risk (RR) with a 95% confidence interval (CI 95%) in the number of participants in each group for each trial. We used the Mantel-Haenszel model to show the effect of the nicotine electronic cigarette as the binary variable and the inverse variance model for the continuous variable. The significance cut-off is *p* < 0.05.

The results from the binary variables were expressed as relative risk (RR) with a confidence interval (abstinence, reduction in consumption of > 50%, occurrence of serious adverse events). The results from the continuous variables (consumption per day) were expressed as a difference from the initial consumption +/- standard deviations in mean difference (MD).

In terms of effectiveness, a calculated relative risk higher than 1 was considered favorable. In terms of safety, a calculated relative risk lower than 1 was in favor of a less toxic effect of the nicotine electronic cigarette. The difference on average is significant for a positive value excluding 0.

The heterogeneity between studies was assessed using the I^2^ statistic. If the I2 value was > 50%, the heterogeneity was considered substantial; it was moderate for values between 25 and 50%; it was low for < 25%.

## Results

**Figure 1:**
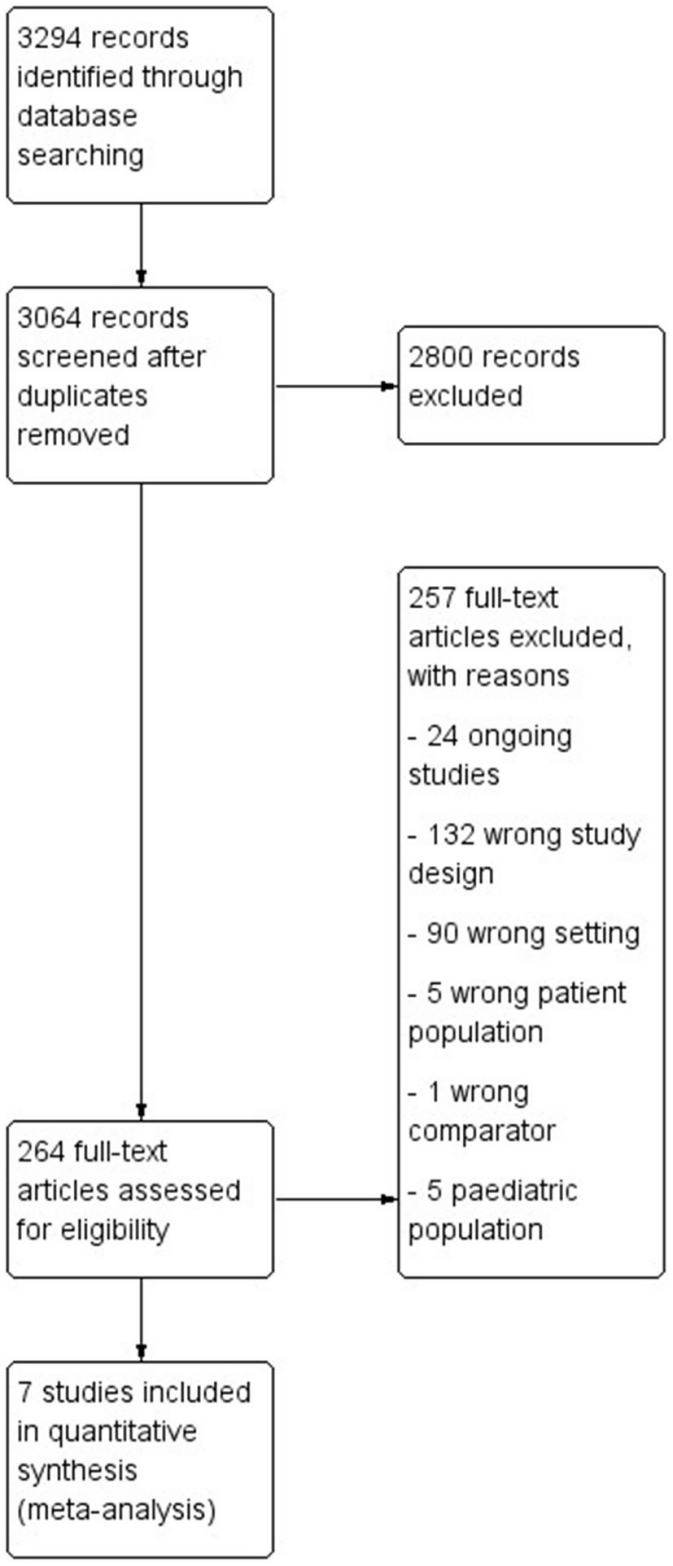
Preferred Reporting Items for Systematic Reviews and Meta-Analyses (PRISMA) flow diagram.

We identified 3,294 articles using our search strategy. After the removal of duplicates and screening titles and abstracts, 264 full texts were assessed for eligibility, but 257 references were excluded mainly due to the lack of outcome data, their having inappropriate study designs or being ongoing studies (Figure 1).

Finally, 7 randomized controlled trials were included in the qualitative analysis (Table 1). They concern smokers with or without the desire to quit. The intervention is the use of the 1^st^ (21, 22) or 2nd generation (23–27) nicotine electronic cigarette. The control group makes use of a patch and other nicotine substitutes, a non-nicotine electronic cigarette or both.

**Table 1:** Characteristics of included studies.

**Authors**	**Study design**	**Duration of intervention: follow up (weeks)**	**Population (*N*)**	**Intervention (*N*)**	**Control (*N*)**	**Outcomes**	**Results**
Caponnetto et al. ECLAT study (21) Italy	RCT 3 arms: 2 intervention groups and 1 control group	12: 52	300 smokers Not intend to quit	1^st^ generation - E cig Arbi Group^®^, ad libitum use, 12 weeks at 7.2 mg (100) - E cig Arbi Group^®^, ad libitum use, 6 weeks 7.2 mg then 6 weeks 5.4 mg (100)	E cig 0 mg (100)	- Abstinence at 12months (since previous visit at 6 months, confirmed with CO < 7 ppm) - Reduction: CPD decrease ≥50% of initial - AE at each study visits	- Significant abstinence in nicotine group at week 12 and 52 vs E cig 0 mg - No statistical difference for smoking reduction - No serious AE reported
Cobb et al. (23)	RCT 4 arms: 2 intervention groups and 2 control group	24:36	520 smokers Not intend to quit	2^nd^ generation - E cig EGO 8mg - E cig EGO 36 mg	- Cigarette substitute - - E cig 0 mg	−7DPP and 28 day or more abstinence with CO < 10 ppm - Reduction: CPD decrease - AE	- Significantly more participants in the 36 mg/ml group than in the 0 mg/ml group are abstinent at 24 weeks - Significant decrease of CPD over times - Serious AE frequency similar across groups, not related to product use
Bullen et al. ASCEND study (22) New Zealand	RCT 3 arms: 2 intervention groups and 1 control group	12: 24	657 smokers Intend to quit	1^st^ generation E cig 16 mg Elusion^®^ (289)	- Nicotine patch 21 mg (295) - - E cig 0 mg (73)	- Continuous abstinence (≤ 5 cigarettes allowed) with CO < 10 ppm - Reduction: CPD decrease ≥50% of initial - AE	- No significant difference between nicotine e cig vs patches and vs 0 mg for abstinence - Significant decrease of CPD at 24 weeks - No serious AE classified as being related to product use
Eisenberg et al. (25) Canada	RCT 3 arms: 1 intervention group and 2 control groups	12: 52	376 smokers Intend to quit	2^nd^ generation E cig 15 mg NJOY^®^ (128)	- E cig 0 mg (127) - Counseling (121)	- 7 day PP abstinence - Continuous abstinence with CO < 10 ppm - Reduction: CPD decrease - AE at each study visits	- No significant differences in abstinence between nicotine and non-nicotine e-cigarettes groups at 12 weeks or 24 weeks - Significant decrease of CPD at 24 weeks - No serious AE
Hajek et al. (28) UK	RCT 2 arms: 1 intervention groups and 1 control group	12 (4 first weeks with behavioral support): 52	884 smokers Intend to quit	2^nd^ generation E cig 18 mg Aspire^®^ (438)	Nicotine replacement group: choice among the range of nicotine replacement products (patch, gum,...) (446)	- Continuous abstinence (≤ 5 cigarettes allowed) with CO < 8 ppm - AE at each study visits	- Significantly more abstinence in the E cig 18mg group than in the NRT group - No serious AE classified as being related to product use
Lee et al. (26) Korea	RCT 2 arms: 1 intervention group and 1 control group	12: 52	150 smokers Intend to quit	2^nd^ generation E cig eGO-c ovale^®^ 0,01 mg/mL (75)	Nicotine gum 2 mg (75)	- Continuous abstinence with CO < 10 ppm + 7 day PP abstinence at 12 and 24 weeks - Smoking reduction - AE	- No significant statistical difference at 12 and 24 weeks for abstinence - Smoking reduction was higher in the nicotine e cigarette group than NRT group - No serious AE reported
Lucchiari et al. (27) Italy	RCT 2 arms: 1 intervention group and 1 control group	12: 52	210 smokers Intend to quit	2^nd^ generation E cig 8 mg (70)	- E cig 0 mg (70) - Counseling (70)	- Continuous abstinence with CO < 7 ppm - Reduction: CPD decrease - AE	- No significant statistical difference after 24 weeks for abstinence - Significant effect of group E cig 8 mg on CPD: after 24 weeks, participants in the nicotine e-cigarette group smoked fewer cigarettes than any other group. - No serious AE reported

RCT, randomized controlled trial; CPD, cigarettes per day; 7 day PP abstinence, 7 day point prevalence abstinence; AE, Adverse events.

### Risk of bias

**Figure 2:**
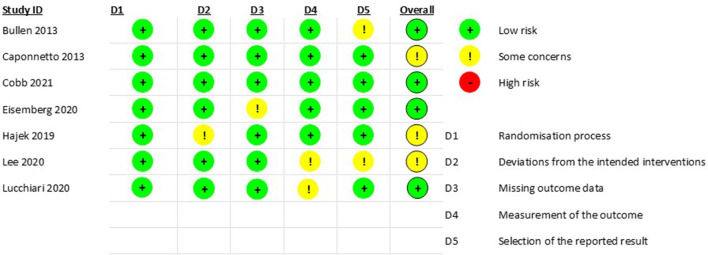
Risk of bias of included studies.

Of all the studies, 4 of them use an open-label arm (Figure 2).

### Intervention effect

The results of the analyses in the form of Forest Plot are listed for online-only supplements.

a) Smoking cessation

After statistical analysis, abstinence at the end of the intervention and follow-up period was significantly higher in the nicotine electronic cigarette group than in the non-nicotine electronic cigarette group, respectively: RR/ 1.97 [1.32, 2.93] and RR: 1.66 [CI 95%: 1.01–2.73] (Table 2). The nicotinic electronic cigarette is significantly more effective than nicotine replacement therapy, with a RR of 1.37 [CI 95%: 1.18–1.59] and 1.49 [CI 95%:1.14–1.95] at the end of the intervention and follow-up period, respectively (Table 2) (Additional file 1).

b) Smoking reduction

**Table 2:** Summary of findings.

**Comparison**	**Abstinence**		**Reduction**		**Cigarette consumption**		**SAE**
	**End of intervention**	**Follow-up**	**End of intervention**	**Follow-up**	**End of intervention**	**Follow-up**	**Follow-up**
E-cigarette vs. placebo	**RR: 1.97 [1.32, 2.93] I2: 33%**	**RR: 1.66 [1.01, 2.73]** **I2: 0%**	RR: 1.22 [0.78, 1.92] I2: NA	**RR: 1.31 [1.02, 1.68]** **I2: 0%**	**MD: 2.97[1.38, 4.57] I2: 89%**	**MD: 1.39 [0.30, 2.48]** **I2: 0%**	RR: 1.22 [0.54, 2.78] I2: 0%
E-cigarette vs. NRT	**RR: 1.37[1.18–1.59] I2: 53%**	**RR: 1.49 [1.14, 1.95]** **I2: 70%**	**RR: 1.48 [1.04, 2.10] I2: 51%**	**RR: 1.47 [1.18, 1.82]** **I2: 0%**	**MD: 1.69 [1.63-1.76] I2: 95%**	MD: 0.81 [0.00 – 1.61] I2: 84%	**RR: 1.53 [1.02, 2.30] I2:13%**

SAE, Serious adverse effects; RR, Risk ratio; NRT, Nicotine replacement therapy; MD, Mean difference/Risk Ratio (M-H, Fixed, 95% CI)/Mean Difference (IV, Fixed, 95% CI)/Bold: p ≤ 0.05/.

We found a significant reduction in consumption > 50% of the baseline with nicotine electronic cigarettes *vs*. nicotine replacement therapy at the end of the intervention and follow-up period [RR: 1.48 (CI 95%: 1.04–2.10) and RR: 1.47 (CI 95%: 1.18–1.82)]. Compared to the non-nicotine electronic cigarette, the nicotine electronic cigarette had a significant effect at the end of the follow-up period [RR: 1.31 (CI 95%: 1.02–1.68)]. The difference in mean daily consumption is significant in the 2 stages of analysis *vs*. non-nicotine electronic cigarettes and only at the end of the intervention *vs*. NRT (Table 2) (Additional file 1).

c) Serious adverse effects

In terms of safety, none of the included studies reports significantly higher serious adverse effects (SAEs) in the nicotine electronic cigarette group. After statistical analysis, compared to NRT, nicotine electronic cigarette has more frequent SAEs but no significant difference is shown with the non-nicotine electronic cigarette [RR: 1.53 (CI 95%: 1.02, 2.30) and RR: 1.18 (CI 95%: 0.65, 2.16)] (Additional file 1). No serious adverse effects directly connected to the use of nicotine electronic cigarettes were reported.

## Discussion

This meta-analysis, exploring the duration of nicotine electronic cigarette effect in the treatment of tobacco use disorders, finds a significant effect on abstinence in 1,618 smokers compared to nicotine replacement therapy and 1,447 smokers in the non-nicotine electronic cigarette condition. With regard to reduction in consumption, the nicotine electronic cigarette is significantly more effective than nicotine substitutes, at the end of the intervention and the follow-up period, and the non-nicotine electronic cigarette, at the end of the follow-up period. No serious long-term adverse effects attributable to the nicotine electronic cigarette were reported in the studies.

Some limits of our study should be acknowledged. First, the interpretation is limited by the small number of studies and patients included in the analysis overall. The data are incomplete for smoking reduction outcomes.

The experimental designs of the trials diverge. Their characteristics remain heterogeneous, particularly in their inclusion criteria. The co-morbidities and the presence of co-addictions differ between the ECLAT and ASCEND studies (21, 22). The secondary analysis of the Bullen study carried out by O'Brien in 2015 found no statistical difference in patients with or without mental illness (29). The majority of studies had a 52-weeks follow-up except for 2 studies of 24 and 36 weeks.

Also, one can bring out the argument that the randomized controlled studies were carried out with electronic cigarettes of different brands, dosages and generations, with an impact on withdrawal symptoms. The ASCEND and ECLAT studies use first-generation electronic cigarettes that distribute nicotine poorly and may have a negative impact on the results. The other studies use second generation devices that have a more efficient nicotine delivery (30). The novel cartridge Pods electronic cigarettes were not evaluated in our review, these new products are emerging in adolescent and young adults (31). They use a nicotine salt rather than freebase nicotine that allow an increase of nicotine concentration into the cartridge. For exemple, Juul products (59 mg nicotine/ml) have a pharmacokinetic profile close to the cigarettes. This pharmacokinetic profile can be dangerous for adolescents and young adults with a higher potential to generate regular use and create a dependence but also can be more efficient for smoking cessation (31, 32).

Similarly, the distribution and support offered to patients differ between these studies. The ECLAT study does not provide any support for withdrawal assistance: no motivational interviewing or cognitive therapy. The ASCEND study offered telephonic support and assistance, while the latest clinical trial in 2019 allowed participants to participate in multiple interviews and face-to-face sessions, which can increase the effect (33). Also, only the ECLAT study, unlike the other studies, included patients who did not intend to quit smoking (21).

Concerning outcome criteria, the definition of abstinence differs between studies. For reduction criteria, there is currently no consensus on a relevant verification method, making it a purely declarative value. The daily consumption was difficult to evaluate due to the lack of data expressed as a reference value. For smoking reduction, two studies (22, 24), excluded patients consuming < 5 cigarettes per day from the calculation, which biases the result.

Our meta-analysis is an update of a precedent publication from 2015 (34), which was the first study to analyse the nicotine electronic cigarette effects by contrasting the end of the intervention and the end of follow-up periods with a threshold duration of 6 months. The last update of the Cochrane meta-analysis (18) reported the significant efficacy of the nicotine electronic cigarette *versus* non-nicotine electronic cigarette in terms of cessation and reduction. This effectiveness is determined at 6–12 months but not in the short-term. Moreover, the analyses include the measurement of physiological parameters but no longer include outcomes criteria that can assess the reduction in consumption. This outcome seems to us to be useful for evaluating the real effectiveness for smokers. Our quantitative analysis shows that the nicotine electronic cigarettes improve the smoking reduction and cessation at the end of the intervention and is stable over time. This is reassuring for smokers trying to quit or reduce smoking.

These conclusions remain consistent with the data from the cohorts of Polosa or Adriaens et al. in 2018 (35–37). We note, moreover, that it is also the frequency of its use that determines its effectiveness, as Berry suggests in 2019 (38). Nevertheless, many studies qualify that electronic cigarettes have no significant impact on abstinence. Khalkhoran and Glantz, in 2016 (39) go even further by talking about the negative effect of the electronic cigarette in terms of cessation and reduction, with rates 28% lower among electronic cigarette users. But this meta-analysis remains debatable because it is based on cross-sectional and cohort studies in addition to randomized clinical trials. It also includes longitudinal studies observing exclusive as well as dual uses of the electronic cigarette with tobacco products. The dual use represents a bias because it can be considered a failure in withdrawal. Indeed, this dual consumption reflects the persistent behavioral and social aspects of the addiction (40). It seems important to note that dual users must receive associated support (41). This support can range from minimal counseling to cognitive-behavioral therapy and online aid. The cost-effective advantage of the electronic cigarette together with support – *vs. the* substitutes – have been reported in a recent study (28).

With regard to the safety of the product, the analyses of this review are difficult to interpret and we cannot perform a detailed SAEs analysis. In the Caponneto and Lee studies, no serious adverse events were declared and in the Bullen study, no details was provided about the SAEs reported even if the authors declare that they are not directly connected to the use of nicotine electronic cigarettes. It is important to have more long-term and detailed data on nicotine electronic cigarette safety. In existing literature, adverse effects like coughing, irritation of the upper airways and nausea tend to diminish over time and during long-term exposure to electronic cigarettes (42). Overall, the electronic cigarette contains 6 constituents of concern, such as nicotine if it is present in high doses, carbonyls, volatile components (benzene, toluene), fine particles, metals and bacteria (43). Exposure to these also appears to be greater when using particular flavors or depending on the voltage of the electronic cigarette (44, 45). Nevertheless, the toxic components are present in much smaller quantities than in conventional cigarettes. The nicotine electronic cigarette, therefore, seems to be safer (46–48), even if recent cases cast doubt on this last statement (49). A survey conducted in Illinois reports 53 cases of multiple lung damage (eosinophilic pneumopathy, diffuse alveolar hemorrhage, lipid pneumopathy) that were already described in 2012 (49). It was suspected that the causes were related to the use of electronic cigarettes. The 2019 update from the CDC (Center for Disease Control and Prevention) lists 2,172 cases of lung damage related to the electronic cigarette (EVALI: electronic cigarette vaping associated lung injury), including 42 deaths (50). The analyses highlight a potential relationship between vitamin E acetate, used as an additive in e-liquid with THC (tetrahydrocannabidiol), CBD (cannabidiol), and these lung lesions.

Finally, it seems particularly important to be observant of young people. The use of the electronic cigarette and the consumption of its additives tend to increase over the years, while proof of its safety is still lacking (51). Its use is therefore based more on curiosity about this trendy and customizable product than on its use for smoking cessation purposes (52). Three studies report a potential link between the initiation of electronic cigarette smoking in young non-smokers and subsequent active smoking (53).

Given the lack of consensus regarding the electronic cigarette, new approaches are being developed. Walker et al. have shown that a combination of nicotine patches and nicotine electronic cigarettes could improve the effectiveness of the electronic cigarette (54). A French study, the ECSmoke study (55) by Doctor Ivan Berlin, is underway and compares the nicotine electronic cigarette to varenicline. We should also mention the Swiss study, ESTxENDS (56), which compares the effectiveness of the electronic cigarette with support *vs*. support alone. Additionally, there are also other studies worth mentioning which explore the different effects of the electronic cigarette (57–59).

This meta-analysis shows the effectiveness of the nicotine electronic cigarette *vs*. non-nicotine electronic cigarette and NRT on smoking cessation and reduction at short-term and is globally stable over time without clear serious side effects. However, there have only been few studies carried out, which does not allow for an affirmation and recommendation of practice. Additional studies with long-term follow-up and new combined treatment seem necessary to confirm the effectiveness of the electronic cigarette. In addition, there are still uncertainties about the risks of its long-term use and its potential role as a gateway into smoking, particularly among young people.

## Data availability statement

The raw data supporting the conclusions of this article will be made available by the authors, without undue reservation.

## Author contributions

PV, AB, and CL-C contributed to the conception and design of the study, overviewed the conduct of the project, participated in the interpretation of the data, and critical revision of the manuscript. ND contributed to conception of research equation and design of the study. SK, PD, PC, MB, and NJ contributed to interpretation of the data and revision of the manuscript. All authors contributed to the article and approved the submitted version.

## Conflict of interest

The authors declare that the research was conducted in the absence of any commercial or financial relationships that could be construed as a potential conflict of interest.

## Publisher's note

All claims expressed in this article are solely those of the authors and do not necessarily represent those of their affiliated organizations, or those of the publisher, the editors and the reviewers. Any product that may be evaluated in this article, or claim that may be made by its manufacturer, is not guaranteed or endorsed by the publisher.
