# Supplementary material for: Duration of the effectiveness of nicotine electronic cigarettes on smoking cessation and reduction: Systematic review and meta-analysis
Source: Front Psychiatry. 2022 Aug 4;13:915946. doi: 10.3389/fpsyt.2022.915946 (PMC9386078; doi:10.3389/fpsyt.2022.915946)
Supplement: Supplementary file 1 [file Data_Sheet_1.docx]

**Additional** **file 1: Forest Plots**

**eFigure 1: Versus Non-nicotine electronic cigarette– End of intervention – Smoking cessation**

**eFigure 2: Versus NRT – End of intervention – Smoking cessation**

**eFigure 3: Versus Non-nicotine electronic cigarette– Long term – Smoking cessation**

**eFigure 4 : Versus NRT – Long term – Smoking cessation**

**eFigure 5 : Versus Non-nicotine electronic cigarette– End of intervention – Smoking reduction**

**eFigure 6 : Versus NRT – End of intervention – Smoking reduction**

**eFigure 7 : Versus Non-nicotine electronic cigarette– Long term – Smoking reduction**

**eFigure 8 : Versus NRT – Long term – Smoking reduction**

**eFigure 9 : Versus Non-nicotine electronic cigarette– End of intervention – Cigarette consumption**

**eFigure 10 : Versus NRT – End of intervention – Cigarette consumption**

**eFigure 11 : Versus Non-nicotine electronic cigarette– Long term – Cigarette Consumption**

**eFigure 12 : Versus NRT – Long term – Cigarette Consumption**

**eFigure 13 : Versus Non-nicotine electronic cigarette- Long term - Serious adverse effects**

**eFigure 14 : Versus NRT- Long term - Serious adverse effects**

**eFigure 1 : Versus Non-nicotine Electronic cigarette – End of intervention – Smoking cessation**

**
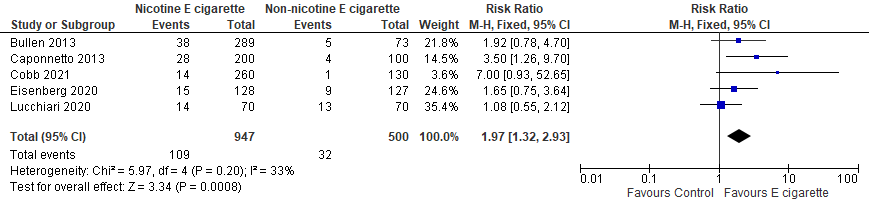
**

**eFigure 2 : Versus NRT – End of intervention – Smoking cessation**


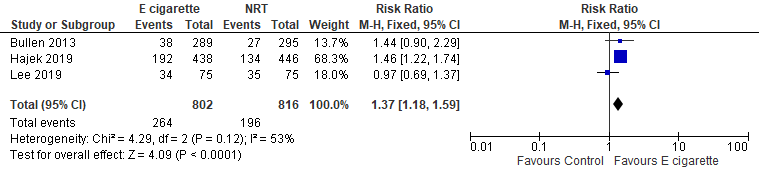


**eFigure 3 : Versus Non-nicotine Electronic cigarette – Long term – Smoking cessation**

**
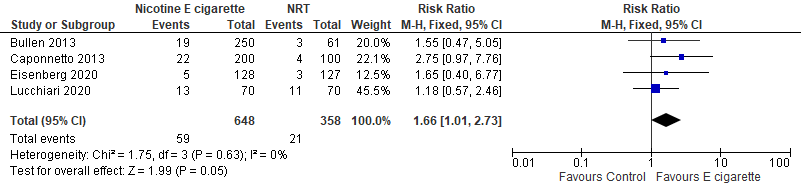
**

**eFigure 4 : Versus NRT – Long term – Smoking cessation**

**
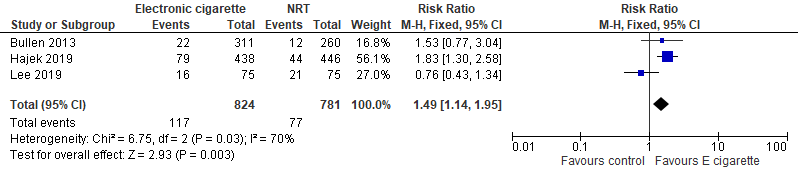
**

**eFigure 5 : Versus Non-nicotine Electronic cigarette – End of intervention – Smoking reduction**

**
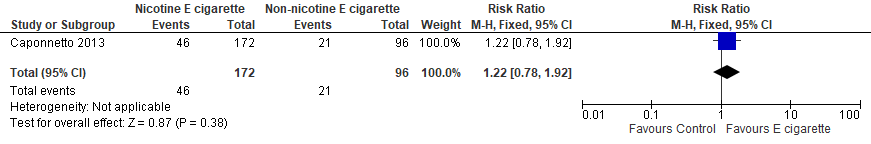
**

**eFigure 6 : Versus NRT – End of intervention – Smoking reduction**

**
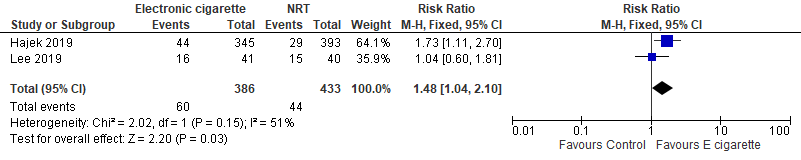
**

**eFigure 7 : Versus Non-nicotine Electronic cigarette – Long term – Smoking reduction**

**
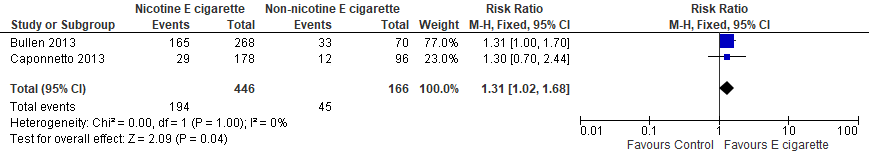
**

**eFigure 8 : Versus NRT – Long term – Smoking reduction**

**
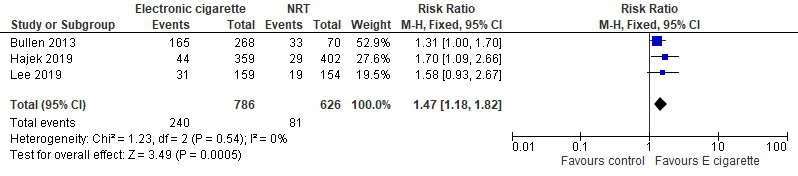
**

**eFigure 9 : Versus Non-nicotine Electronic cigarette – End of intervention – Cigarette consumption**

**
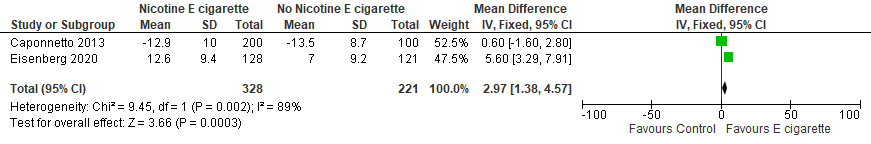
**

**eFigure 10 : Versus NRT – End of intervention – Cigarette consumption**

**
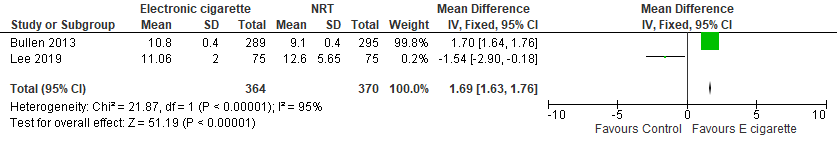
**

**eFigure 11 : Versus Non-nicotine Electronic cigarette – Long term – Cigarette Consumption**

**
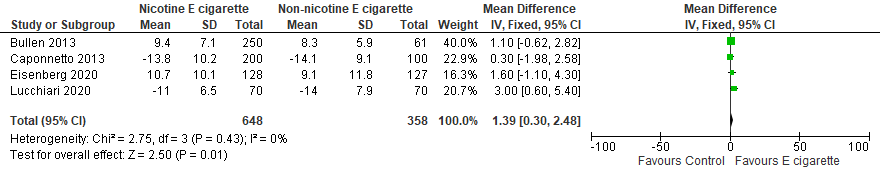
**

**eFigure 12 : Versus NRT – Long term – Cigarette Consumption**

**
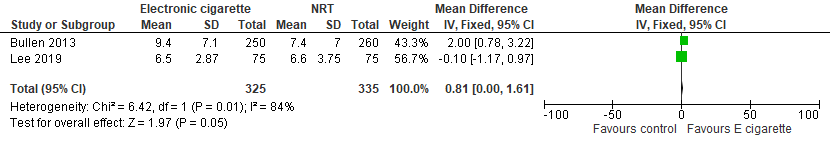
**

**eFigure 13: Versus Non-nicotine Electronic cigarette - Long term - Serious adverse effects (ITT analysis)**

**
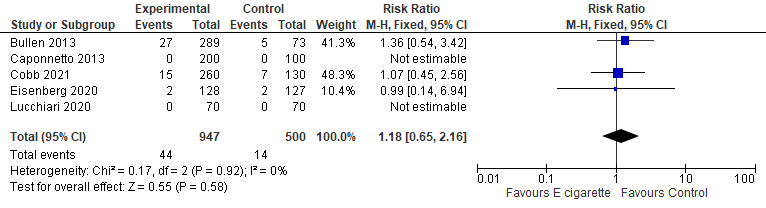
**

**eFigure 14 :Versus NRT- Long term - Serious adverse effects (ITT analysis)**

**
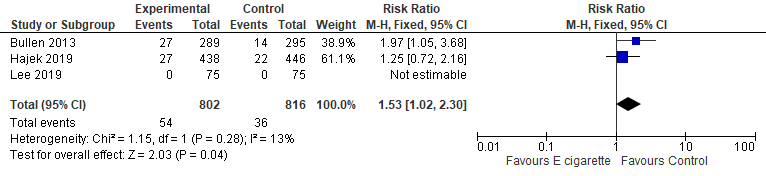
**
